# Role of Scheimpflug imaging in the diagnosis and management of keratitis caused by caterpillar seta

**DOI:** 10.4103/0974-620X.71900

**Published:** 2010

**Authors:** Ozgur Bulent Timucin, Mehmet Baykara

**Affiliations:** Department of Ophthalmology, Van Education and Research Hospital, Van/Turkey; 1 Department of Ophthalmology, Uludag University School of Medicine, Bursa/Turkey

**Keywords:** Caterpillar seta, keratitis, lepidoptera, ophthalmia nodosa, scheimpflug imaging.

## Abstract

A 16-year-old boy presented with a history of an accidental hit to the left eye by a butterfly (*Lepidoptera*). One seta fragment was found to be embedded into the cornea and inflammation secondary to penetration of caterpillar seta was seen around the seta fragment. Scheimpflug imaging was performed in the area showing caterpillar seta. Corneal infiltration was imaged as a hyper-reflective area. Lesion dimensions were measured with calipers. Scheimpflug imaging is a potential tool for localization of corneal lesions, monitoring the progress of the injury and evaluating the treatment response objectively.

## Introduction

**Figure 1:**
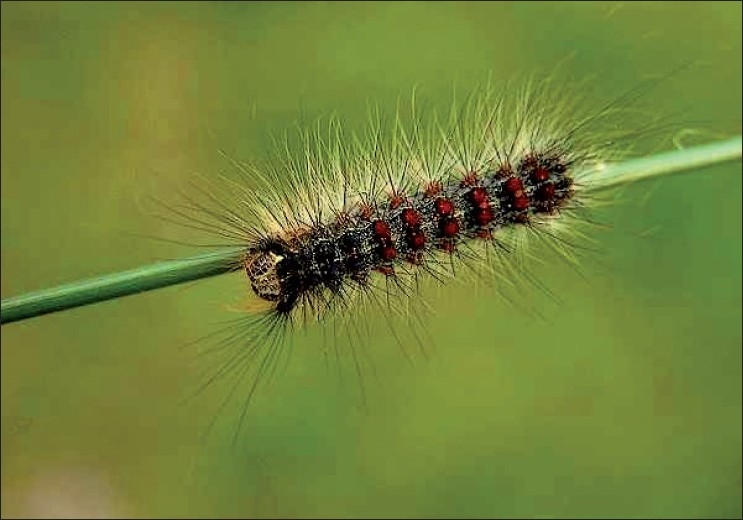
Caterpillar setae. (modified from Dessy R. Hairy’ Insects and Spiders Spurs, Spines, Setae, and Sensilla. Micscape Magazine. 2009 Jan;159. with permission)

Caterpillars are the larval form of a member of the insect order *Lepidoptera*, which includes butterflies and moths [Figure 1]. Ophthalmia nodosa is the term used to describe ocular lesions caused by caterpillar seta,[1] the most common type of insect eye injury. Diagnosis, treatment and follow up of corneal involvement, as assessed with the slit-lamp and less commonly with the aid of serial anterior segment photography, which involves the assessment of the depth and the extent of pathologic features, is subjective and depends significantly on the experience of the examiner and is usually based on the clinical features of the corneal epithelial defect and stromal infiltration.

Herein is the first report that describes the importance of Scheimpflug imaging in the diagnosis and management of keratitis due to caterpillar seta.

## Case Report

**Figure 2:**
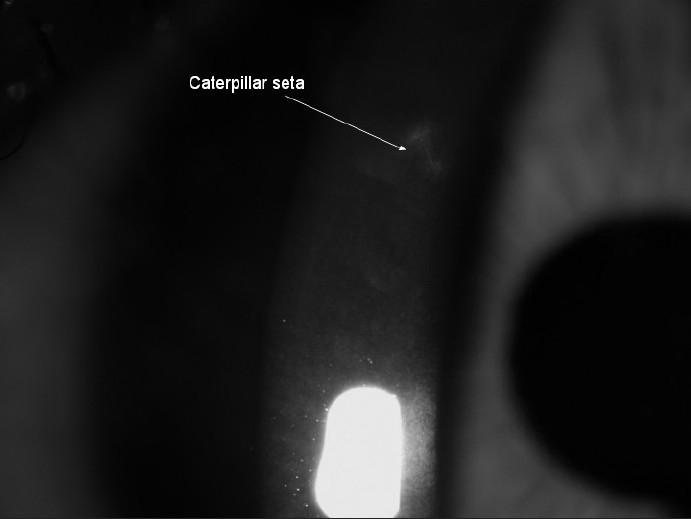
Slit-lamp photograph of left eye showing caterpillar seta (arrow)

**Figure 3:**
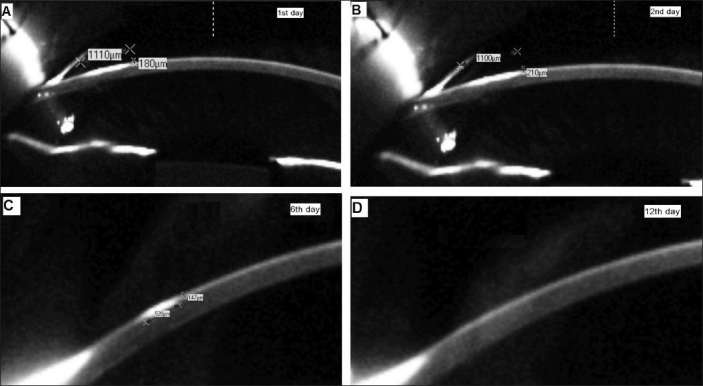
Scheimpflug imaging showing area of keratitis caused by caterpillar seta. A) Scheimpflug imaging obtained at presentation showing corneal infiltration with dimensions 1110 μm in width and 180 μm in depth. B) Imaging obtained two days after presentation showing nearly no changes in dimensions. C) Scheimpflug imaging obtained six days after first presentation showing reduction of infiltration. D) Twelve days after first presentation. The infiltration had healed completely and resolved without scarring

A 16-year-old boy presented with a history of an accidental hit to the left eye by a butterfly (**Lepidoptera**) while driving a bicycle, following which he started having conjunctival injection. He was referred to us after 15 days of follow-up by a local ophthalmologist because of persistent conjunctival injection and foreign body sensation. His chief complaint was foreign body sensation. Visual acuity was 20/20 in the affected eye. On slit-lamp examination, the anterior chamber had no cellular reaction and funduscopic examination was unremarkable. Intraocular pressure was 16 mm-Hg by applanation. One seta fragment was found to be embedded into the cornea and inflammation secondary to penetration by the caterpillar seta was seen around the seta fragment [Figure 2]. The epithelium was intact with negative fluorescein staining. Scheimpflug imaging (Pentacam 70700: Oculus, Wetzlar, Germany) showed a single, high-reflective site, suggestive of the caterpillar seta in the superficial cornea at 10:00 o’clock position, irregular stroma and interrupted endothelial surface integrity [Figures 3a and b]. There was also localized corneal thickening in this region [Figures 3a and b]. Exposed foreign body was removed with a forceps and identified as seta. After removal of the seta under a microscope, the inflammation subsided. Initial Scheimpflug imaging showed that the infiltrate was 1110 μm in width and 180 μm in depth [Figure 3a]. The patient was then prescribed topical ciprofloxacin and fluorometholone eye drops four times daily, which was then tapered gradually over the next two weeks. One week later, the conjunctival injection was reduced. On Scheimpflug imaging, the dimension of the corneal lesion was reduced to 528 μm in width and 147 μm in depth [Figure 3c]. Twelve days later, the infiltration had healed completely and resolved without scarring. At follow-up, Scheimpflug imaging showed that the superficial high reflective site disappeared, the stroma had gained its regularity and the integrity of corneal endothelial surface was visible [Figure 3d]. The eye was free of inflammation at three weeks’ follow-up.

## Discussion

Contact with caterpillar setae can be through direct contact with caterpillars, the larval coccoon into which setae may be shed and interwoven, with adult *Lepidoptera* that may carry larval setae on their bodies, and direct exposure to adult and wind-borne setae.[1] The *Lepidoptera* larva’s urticating hair has poison glands under the setal base. When seta is ruptured by trauma, the poison causes an allergic reaction in the involved area. In such cases, it is necessary to remove all insect parts from the human body.[2] Surgical intervention may not be required for patients with no symptoms or clinical signs of intraocular inflammation.[3]

The spectrum of ocular pathology was classified by Cadera *et al*,[4] : Type 1- Acute toxic reaction with chemosis, inflammation, epiphora, and foreign-body sensation. Type 2- Chronic mechanical keratoconjunctivitis (hairs in bulbar/palpebral conjunctiva). Type 3- Grey yellow nodules (granulomas) under conjunctiva. Type 4- Iritis and hairs in anterior chamber and Type 5- Vitreoretinal involvement. Based on this classification, majority of the reported patients belong to Types 1 or 2.[5,6] The suggested treatment modalities based on the degree of involvement is as follows: Types 1 and 2 - Irrigation followed by meticulous removal of setae. Topical antibiotics and steroids. Type 3- Surgical excision of nodules. Type 4- Topical steroids-iridectomy for nodules or operative removal of setae, and Type 5- Local or systemic steroids. Resistant cases-vitrectomy with removal of setae. Our patient had type 2 involvement and was managed accordingly. Even though insect induced ocular complications, such as iritis, conjunctival or iris granulomas, vitritis[7] and endophthalmitis[8,9] have been reported, our patient showed good clinical course without complication. Based on our case, we recommend careful examination of the cornea for setae in patients with unilateral redness. In patients with unilateral persistent conjunctival injection and foreign body sensation and associated localized corneal infiltrate, the clinician should suspect the presence of a foreign body in the cornea. Caterpillar hairs can cause corneal infiltrates and should be considered in the differential diagnosis.

In clinical practice, imaging of the corneal lesions has traditionally been carried out with slit lamp biomicroscopy. However, objective quantitative assessment of anterior segment structures with it is limited. Scheimpflug imaging in ophthalmology is a technique that allows the assessment of the anterior segment of the eye from the front of the cornea to the back of the lens in coronal plane. It captures slices through the anterior chamber to provide a fast, accurate and objective documentation of the anterior segment.[10] Scheimpflug imaging has been used extensively to examine intraocular lens decentration, tilt, anterior capsule contraction, posterior capsule opacification, anterior and posterior elevation maps, curvature, pachymentry, anterior chamber depth, chamber volumes and surface-derived Zernike polynomials. Besides, as presented in this report, Scheimpflug analysis with calipers enables ophthalmologists to quantify the corneal inflammatory changes and gauge response to treatment.

Our early observation suggests that calipers with Scheimpflug imaging are available to measure both the depth and size of the area of infiltration in cases with keratitis due to caterpillar setae. Scheimpflug imaging is a potential tool for localization of keratitis in the cornea, monitoring the progress of the injury and provides an objective basis for better patient counseling.
